# Exploring the Impact of Localized COVID-19 Events on Intercity Mobility during the Normalized Prevention and Control Period in China

**DOI:** 10.3390/ijerph192114421

**Published:** 2022-11-03

**Authors:** Mingke Xie, Yang Chen, Luliang Tang

**Affiliations:** 1 State Key Laboratory of Information Engineering in Surveying, Mapping and Remote Sensing, Wuhan University, 129 Luoyu Road, Wuhan 430079, China; 2 Beidou Research Institute, Faculty of Engineering, South China Normal University, Foshan 528000, China

**Keywords:** intercity travel, COVID-19, policy index, localized events, gravity model

## Abstract

Uncontrolled, large-scale human mobility can amplify a localized disease into a pandemic. Tracking changes in human travel behavior, exploring the relationship between epidemic events and intercity travel generation and attraction under policies will contribute to epidemic prevention efforts, as well as deepen understanding of the essential changes of intercity interactions in the post-epidemic era. To explore the dynamic impact of small-scale localized epidemic events and related policies on intercity travel, a spatial lag model and improved gravity models are developed by using intercity travel data. Taking the localized COVID-19 epidemic in Xi’an, China as an example, the study constructs the travel interaction characterization before or after the pandemic as well as under constraints of regular epidemic prevention policies, whereby significant impacts of epidemic events are explored. Moreover, indexes of the quantified policies are refined to the city level in China to analyze their effects on travel volumes. We highlight the non-negligible impacts of city events and related policies on intercity interaction, which can serve as a reference for travel management in case of such severe events.

## 1. Introduction

The outbreak of the novel coronavirus pandemic (COVID-19) has severely threatened global public health and socioeconomic development [1,2]. The public health policies (such as restricting travel and keeping social distance) were emergently implemented to reduce the mobility of people [3,4,5]. Fortunately, after more than two years of joint efforts of mankind, the COVID-19 epidemic has been effectively controlled. As one of the most stringent countries in epidemic prevention and control, China has entered a period of normalized epidemic prevention and control (NEPC) from global static management, and the distribution of epidemic situations in China has also narrowed from the national scope to local areas. However, it is undeniable that localized epidemics can also cause substantial disruption to people’s lives, so it also requires our focused attention. 

Intercity travel, which reflects the mass movement of people, has always been one of the key points in the study of human mobility. Abundant achievements have been made in the research of intercity travel, including the analysis of spatial and temporal patterns of intercity travel [6,7,8], flow prediction [9,10], influencing factors analysis [5,9,10], etc. In the aftermath of the COVID-19 pandemic outbreak, there emerged numerous articles exploring the relationship between the pandemic and human activities. On the one hand, scholars have analyzed the relationship between the objective development state of the epidemic, subjective risk attitudes of the public and mobility responses. The results of Chan et al. [11] show that risk-taking attitudes are a critical factor in predicting reductions in human mobility and social confinement around the globe. On the other hand, scholars have, in turn, analyzed the impact of travel behavior on the spread of the epidemic and the changes caused to the psychology of the population. Many of these issues have been explored in intercity travel studies. For instance, studies have shown that population movement greatly changes the spatial and temporal patterns of the epidemic [5,12,13,14]; restricting intercity mobility and enforcing intracity social isolation are effective in reducing the viral transmission of COVID-19 [15,16,17,18]. In particular, intercity high-speed rail and flights have had a significant impact on the spread of COVID-19 [19,20,21]. Further studies have conducted a more detailed analysis by differentiating between different travel ranges, travel purposes, or travel modes [21,22,23]. These studies quantify the effectiveness of travel restrictions on delaying the epidemic growth and limiting the spread scope of COVID-19 in China, contributing to understanding how intercity mobility affects the transmission of COVID-19. Meanwhile, the relationship between nonpharmaceutical interference measures (i.e., governmental policy measures in dealing with the epidemic event) and the spread of the diseases in various countries has also been studied by many scholars in studies on the COVID-19 epidemic [24,25,26,27]. The widely accepted conclusion is that governmental interference, especially travel restrictions, has a significant inhibitory effect on reducing the spread of the pandemic. This corresponds to the previously mentioned studies on the relationship between COVID-19 and human mobility.

In general, most of the existing articles focus on large-scale epidemics, attributing the spread of infectious diseases mainly to population mobility and attributing the stagnation of human activities mainly to policy interventions and group cognition. Focusing on the first-round global pandemic in 2020, they access the potential risk and scope of transmission from travel [28], and intuitively demonstrate a significant negative relationship between the severity of COVID-19 and mobility [29,30,31,32]. However, with the gradual receding of COVID-19 pandemic, studies focusing on small-scale epidemics are relatively insufficient, and comparative studies on the relationship between localized epidemics, nonpharmaceutical interventions, and mobility are lacking. Few studies have focused on the influence of variable localized epidemic patterns on mobility during the period of NEPC one or two years after the initial outbreak.

As a result, our purpose is to clarify changes in intercity travel patterns during the NEPC periods in China. On this basis, the dynamic effects of localized epidemic events and policies on intercity travel intensity are quantitatively explored. In order to explain our research objectives more clearly, we break them down into the following three questions based on existing articles [28,33,34] exploring large-scale epidemics in 2020: (1) Could small-scale localized outbreaks affect intercity travel? (2) What is the impact scope of localized epidemic on crowd intercity travel? (3) How do we quantificationally assess the impact of localized epidemics and corresponding epidemic prevention policies on intercity travel? To better answer these three questions, we synthesize traditional static urban attributes such as population, economy, and resources into one urban size indicator, and put the focus on two types of dynamic influences: epidemic events and corresponding epidemic prevention policies. At the same time, the factor of spatial relative relationship between cities is brought to the forefront.

In summary, the main contributions of the study are as follows: (1) The changes in Chinese intercity travel patterns before COVID-19 pandemic and during the NEPC period are compared. (2) We enrich the portrayal of epidemic events from a policy perspective and illustrate the significant changes that localized epidemic events may bring to intercity travel qualitatively and quantitatively. (3) We demonstrate that policy index can effectively capture the negative impact of catastrophic events on human mobility in a country or region, and highlight that localized events as well as policies are non-negligible parts of the intercity travel studies.

## 2. Materials and Methods

### 2.1. Study Area

In this paper, 362 cities in China were used as the study area, including 4 municipalities directly under the central government, 331 prefecture-level administrative units, and 27 county-level administrative units in mainland China.

The period from 9 December 2021 to 14 January 2022, was selected as the study period under the general context of NEPC. The study period was divided into three phases with the epidemic development in Xi’an, Shaanxi Province, China as the main line. (1) The period from 9–22 December 2021 is phase P1, which is the initial phase of the outbreak in Xi’an. In phase P1, the first confirmed case was seen in Xi’an on the first day, after which the number of new confirmed cases increased slowly every day, with a few additional cases from cities such as Shaoxing, Ningbo, and Hulunbuir. This is roughly representative of the epidemic situation in China during the period of NEPC. (2) The period from 23 December 2021 to 4 January 2022 is the P2 phase, which is the rapid outbreak phase of the epidemic in Xi’an. The average daily number of new confirmed cases in Xi’an during this time accounts for 91.7% of that nationwide. (3) The P3 phase is from 5 January 2022 to 14 January 2022, during which the number of new cases per day in Xi’an begins to show a decreasing trend. However, due to the aftermath of the epidemic in phase P2, small-scale localized outbreaks occurred in more cities across the country. Accordingly, more local governments began to introduce stricter localized epidemic prevention and control policies.

### 2.2. Intercity Travel Index

The travel data to measure human mobility come from the Baidu Migration Platform, which provides migration data of crowds such as the intracity or intercity travel scale index and intercity travel ratio for 362 cities in China. These data are based on the synthesis of nearly 120 billion location services from more than 1.1 billion cell phones per day in China, whose reliability has been verified by abundant studies [33,35]. We obtained Baidu migration data for a total of 49 days before the epidemic in 2019, during a week without epidemic in Xi’an in December 2020, and during the study period with local epidemic outbreaks in Xi’an, which is the focus of this paper. Then, the daily move-in scale index and move-out scale index of each city are averaged to represent daily intercity travel scale (*ITS*). The magnitude of the change in ITS in the study period P2 relative to P1 is calculated according to the following formula.

(1) $$ITS_{change}=\frac{\frac{1}{N_{P2}}\sum_{j=1}^{N_{P2}}ITS_{jk}-\frac{1}{N_{P1}}\sum_{i=1}^{N_{P1}}ITS_{ik}}{\frac{1}{N_{P1}}\sum_{i=1}^{N_{P1}}ITS_{ik}}*100\%$$

In the equation, $ITS_{change}$ denotes the rate of change in the intercity travel scale for city k in period *P*2 relative to *P*1. $N_{P1}$ and $N_{P2}$ denote the total number of days included in each period, respectively. The result of $ITS_{change}$ can quantificationally reflect whether the localized epidemic in Xi’an brings about a national change in intercity travel.

### 2.3. Epidemic Data and Policy Index

This study expresses the impact of epidemics on travel from two perspectives. The most common and intuitive method is to use epidemic case data, and the other is the government’s epidemic prevention policy. On the one hand, we consider that at the group level, epidemic case data are the main source of fear generated by the public, and that people’s travel willingness decreases as the number of epidemic cases increases. The epidemic prevention policy, on the other hand, is an objective evaluation of the epidemic event after integrating multiple factors.

Day-by-day epidemic case data for each city, including the daily cumulative number of confirmed cases, the cumulative number of cures, the cumulative number of deaths, and new confirmed cases during the study period, are collected from Dingxiang Doctor, Tencent News, and government information disclosure websites of each prefecture-level city in China. The data collection is performed by compiling a web crawler in Python language, followed by data organization and cleaning. The variables and implications are listed in Table 1.

To measure the status of a city’s epidemic itself, the formula was constructed using three indicators: the number of existing confirmed cases (*Existing*), the number of new cases per day (*New*), and the average number of new cases per day in the previous seven days (*7d_avg_new*). Existing confirmed cases as the source of infection are the main cause of new confirmed cases and indicate the potential spread and impact of the epidemic. The number of new cases per day indicates the current level of development of the epidemic. The indicator of average daily additions in the previous seven days then takes into account the lagged effect of the COVID-19 epidemic. The formulas for calculating *Existing* and *Covid_status* are shown in Equations (2) and (3).

(2) Existing=Total−Cures−Deaths

(3) Covid_status=α∗Existing+β∗New+γ∗7d_avg_new

Meanwhile, city-level epidemic prevention policy indices reflecting epidemic development are constructed. The provincial policy control index (*PPI*) is calculated by collecting a series of indices and quantifying various policy measures, which is based on Hale et al. [36,37,38]. The article considers measures such as public transportation closures, mobility restrictions, and cancellation of public gatherings implemented at the provincial level in 31 provincial administrative regions of China (excluding Hong Kong, Macau, and Taiwan). The values of PPI are in the range of 0–100. The detailed calculation formula is as follows.

(4) $$I_{kt}=100\frac{v_{kt}-0.5\left(F_{k}-f_{kt}\right)}{N_{K}}$$

(5) $$PPI=\frac{1}{n}\overset{n}{\underset{k=1}{\sum }}I_{k}$$

In Equation (4), $I_{kt}$ denotes the score of each subcategory of the policy indicator for policy indicator category k in date  t. $N_{K}$ is the maximum value of the indicator and $v_{kt}$ is the value of the policy control index given by a professional evaluation team. $F_{k}$ denotes whether the indicator has a flag ($F_{k}=1$ if the indicator has a flag variable and 0 otherwise). $f_{kt}$ is a binary signifier. Under the condition of $F_{k}=1$, $f_{kt}=0$ indicates that the policy in the region targets a smaller localized area, so an index correction of 0.5 is applied. On the contrary, $f_{kt}=1$ indicates that policy k applies to the whole area of the region. In Equation (5), PPI denotes the province-specific epidemic policy control index, which is obtained by averaging $I_{k}$.

This study is based on a city scale, so further detailed calculations of the city-level policy index (*CPI*) are required, taking into account the different epidemic situations in each city in the province. The number of epidemic risk areas in each city during the period of the study was obtained from the official website of the China Health and Wellness Commission Epidemic Risk Rank Query. Neighborhoods within cities were officially classified into high-risk, medium-risk, and low-risk areas based on the epidemic risk level. Areas other than high and medium-risk zones are considered low-risk, so the changes due to the low risk level are not considered in the formula. The city-level epidemic policy control index (*CPI*) is calculated as follows.

(6) $$CPI=PPI*\left(1+R_{H}m_{H}+R_{M}m_{M}\right)$$

In Equation (6), *CPI* indicates the epidemic policy control index of a single city and PPI indicates the policy control index of the province to which the city belongs. The larger the *CPI* value, the stricter the epidemic prevention measures of the city. $R_{H}$ and $R_{M}$ correspond to the increase of epidemic prevention policy strictness brought by high-risk and medium-risk zones, respectively, and are taken as 0.7 and 0.3 based on real experience. $m_{H}$ and $m_{M}$ represent the number of high and medium-risk zones.

### 2.4. Spatial Lag Model

Considering the spatial dependence of intercity travel, a spatial lag model (SLM) is used to determine the impact of the epidemic in Xi’an on other cities. This can be intuitively understood as a “one-to-many” impact analysis.

The spatial lag model introduces the spatial lag factor of the dependent variable as an explanatory variable and considers the existence of spatial autocorrelation of the dependent variable between adjacent study regions.

(7) $$Y=\rho WY+X\beta +\varepsilon \;\varepsilon \sim N\left[0,\sigma^{2},I\right]$$

In SLM, Y is the N×1-dimensional dependent variable and X is an N×K-dimensional vector containing K explanatory variables. ρ is the spatial autocorrelation coefficient, and if ρ is significant, it indicates that there is some spatial dependence between the dependent variables. WY is the spatial lag factor, β indicates the degree of X’s influence on Y, and ε is the random error term.

In this study, the change in intercity travel scale (see Section 2.2 for details) is used as the dependent variable, and the actual travel distance of Xi’an from other prefecture-level cities (obtained through the official API of Baidu Maps) is used as the explanatory variable.

### 2.5. Measurement of Influencing Factors of Intercity Interaction

The gravity model is one of the most widely used methods in human mobility-related studies because of its good overall fit results and concise parameters [39]. The idea of gravity model is inherited from Newton’s gravitation model, in which there are two main features: mass and distance. In a large number of travel studies, researchers have found through long-term observation that the strength of spatial connection between two cities is proportional to the population size and economic strength between the two places, and decreases with increasing distance, which fits with the idea of gravity model [40]. Therefore, in the field of intercity interaction, gravity model is often used for travel distribution prediction, and is improved to reflect the generation–attraction relationship between the traffic volume at the starting point and the end point of travel. The basic expression form of the gravity model is shown in Equation (8).

(8) $$F_{ij}=g\frac{P_{i}P_{j}}{d_{ij}^{\beta }}$$

In the formula, $F_{ij}$ denotes the magnitude of the gravitational force between city pairs. g is the gravitational coefficient. $P_{i}$ and $P_{j}$ denote the population size of departure city i and destination city j, which is also commonly expressed in terms of the city’s GDP. $d_{ij}$ is the migration cost between city i and j, which can be characterized in the form of linear distance, actual distance, economic distance, etc. This paper inherits the idea of classical gravity model for intercity travel and improves it to analyze the impact of epidemic-related policies on cross-city travel in multiple cities. It can be intuitively understood as a “many-to-many” impact analysis.

There are two common research ideas and improvements in the study of factors influencing intercity travel based on the gravity model. One is to focus on the attributes of the departure city (O) and the arrival city (D) itself. This type of research argues that the city’s own attributes such as population, number of industries, and economic level influence the ability of the city to generate or attract trips. The stronger this ability is, the greater the volume of travel exchange with other cities. Studies tend to improve the fitting effect of gravity model by optimizing those city attribute factors. In order to express the quality of cities more scientifically, with reference to previous research [41], we consider fifteen attributes of cities in a comprehensive manner to express the attractiveness and radiation of travel by the indicator of city size.

The other one focuses on the connection between two cities and elaborates the reasons for passenger flow generation. Scholars often add some constraints to the basic gravity model. For example, Wang et al. [39] introduced a series of improved gravity models that consider the proximity, complementarity, and diversity of industrial structures between cities by adding urban industrial structures as invisible distance constraints on intercity interactions. Whether it is a single-constraint model, a double-constraint model, or others, most of the existing studies consider the aggregate constraints on the amount of departure and arrival at the origin or destination, and rarely consider the constraints on the spatial interactions. The epidemic events and policies are precisely the restrictions on the relationship between travel supply and demand. Because the outflow and inflow are restricted, the travel demand at origin will decrease and the travel attractiveness at destination will also decline. How to express the constraints of epidemic events and policies on intercity travel interactions is a scientific question. Our approach is to impose constraints on the OD flow of travel between two cities in the gravity model. Then, taking the double logarithm of the basic equation, we obtain the following model expression.

(9) $$\mathrm{MODEL}\left(0\right):\;\ln F_{ij}=\lambda_{1}lnP_{i}+\lambda_{2}lnP_{j}+\beta lnD_{ij}+\varepsilon$$

(10) $$\mathrm{MODEL}\left(1\right):\;\ln F_{ij}=\lambda_{1}lnP_{i}+\lambda_{2}lnP_{j}+\beta lnD_{ij}+\delta Covid_{status}+\varepsilon$$

(11) $$\mathrm{MODEL}\left(2\right):\;lnF_{ij}=\lambda_{1}lnP_{i}+\lambda_{2}lnP_{j}+\beta lnD_{ij}+\gamma_{1}lnCPI_{i}+\gamma_{2}lnCPI_{j}+\varepsilon$$

MODEL(0) (Equation (9)) is another mathematical expression of Equation (8), where $\lambda_{1}$ and $\lambda_{2}$ denote the elasticity coefficients of the size of the origin and destination, respectively. β is the distance attenuation index, whose original form takes a fixed value of two, and here we regard it as one of the variables to be solved. Moreover,$\;P_{i}$ and $P_{j}$ are no longer simply measured by the population size of cities but are expressed by the city scale index (*CSI*). $d_{ij}$ denotes the actual distance between two pairs of cities obtained through the Baidu map platform. Further, the epidemic status indicator (*Covid_status*) is added to MODEL(1) as a visual descriptor of the epidemic. *CPI* in MODEL(2) is the municipal epidemic policy control index as the main explanatory variable.

Based on MODEL(2), $SamePro_{ij}$ and $Adjacent_{ij}$ variables are introduced as moderator variables to indicate whether two places are "in the same province" and "adjacent", separately. Both variables take the value of 0 or 1 to check the heterogeneity effect of the core variable. The heterogeneity discussion model is as follows.

(12) $$\mathrm{MODEL}\left(3\right):lnF_{ij}=\lambda_{1}lnP_{i}+\lambda_{2}lnP_{j}+\beta lnD_{ij}+\varepsilon +\gamma_{1}lnCPI_{i}+\gamma_{2}lnCPI_{j}+\gamma_{3}VAR*lnCPI_{i}+\gamma_{4}VAR*lnCPI_{j}$$

where *VAR* refers to $SamePro_{ij}$ and $Adjacent_{ij}$. We mainly examine $\gamma_{1},\;\gamma_{2},\;\gamma_{3}$, and $\gamma_{4}$. If VAR∗lnCPI has a significant effect, the effect of epidemic policy on intercity travel is heterogeneous with the value of VAR.

### 2.6. City Scale Index

As mentioned in the previous section, the gravity model is widely used because of its simplicity and effectiveness. However, the parameters selected by the basic model are too single and the consideration of other factors such as social factors and traffic factors is lacking, which makes the calculation results differ from the actual situation.

City scale (quality) determines the amount of travel radiation that a city can generate and the ability to attract population inflows from other areas. The basic gravity model (Equation (8)), in which city quality is determined by population or GDP alone, is not accurate. City quality is influenced by several factors, and while a city’s population and GDP are extremely important influences, other factors such as the social resources a city can provide, its geographic location, and the city’s industrial layout all influence city quality. Therefore, fifteen statistical indicators characterizing the basic level of development of each city in China are collected to calculate the city scale index (*CSI*). These data are mainly obtained from the 2019 China Statistical Yearbook and the China City Statistical Yearbook, which include five major aspects: population, economy, resources, urban connectivity, and administrative district location. The descriptive statistics of each index are detailed in Table S1.

The principal component analysis (PCA) method was used to calculate *CSI*. PCA method determines the indicator weights by calculating the correlation degree between multiple indicator data and the coefficient of variation of each data, leading to a comprehensive evaluation. It adopts the idea of dimensionality reduction and is able to reduce multiple indicators into a few composite indicators, avoiding multicollinearity among variables under the condition of retaining as much information as possible about the original variables, which is an effective and objective multivariate statistical method [42].

## 3. Results

### 3.1. Changes in the Pattern and Scale of Intercity Travel under Localized Epidemics

To begin with, by processing the Baidu migration flow data, we visualized three periods of intercity travel patterns with a focus on the city of Xi’an, China. These three periods are: (1) the period before the emergence of the COVID-19 outbreaks in 2019, (2) the NEPC period without COVID-19 outbreak in Xi’an city (after the first wave of COVID-19), and (3) the NEPC period with small-scale COVID-19 events in Xi’an city. The OD maps for intercity travel are shown below.

**Figure 1:**
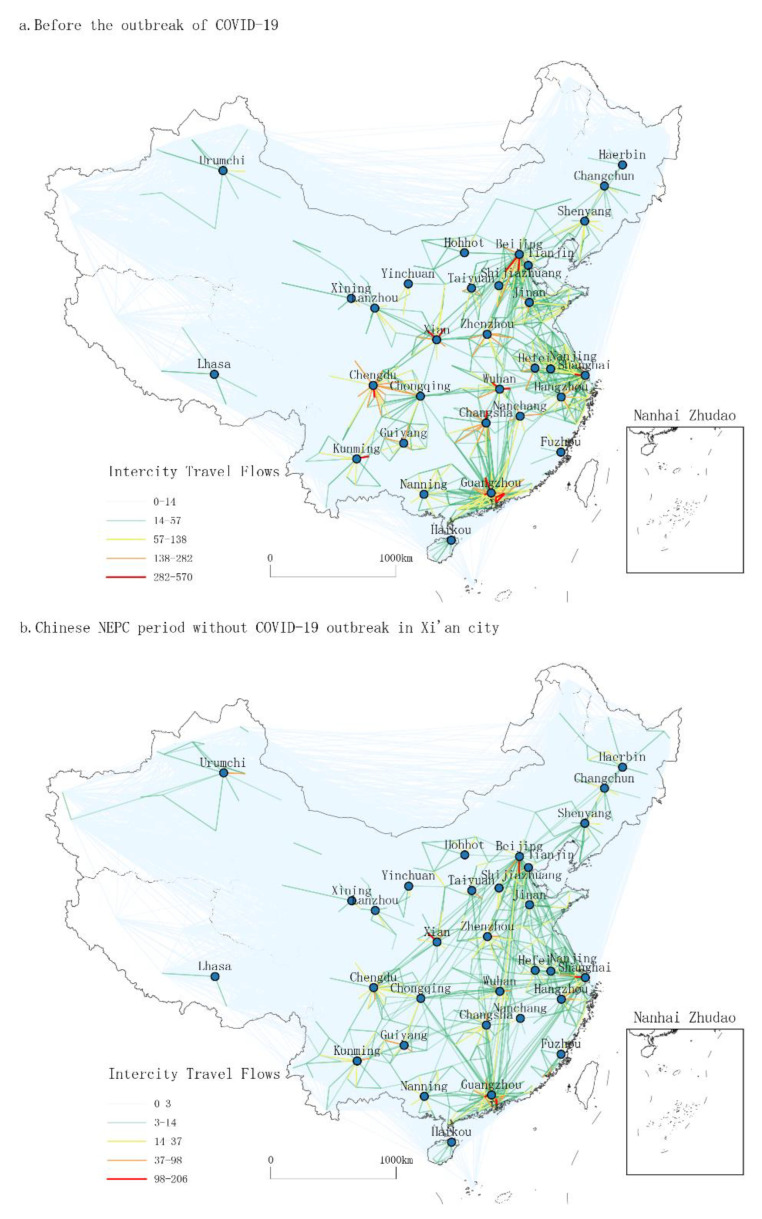

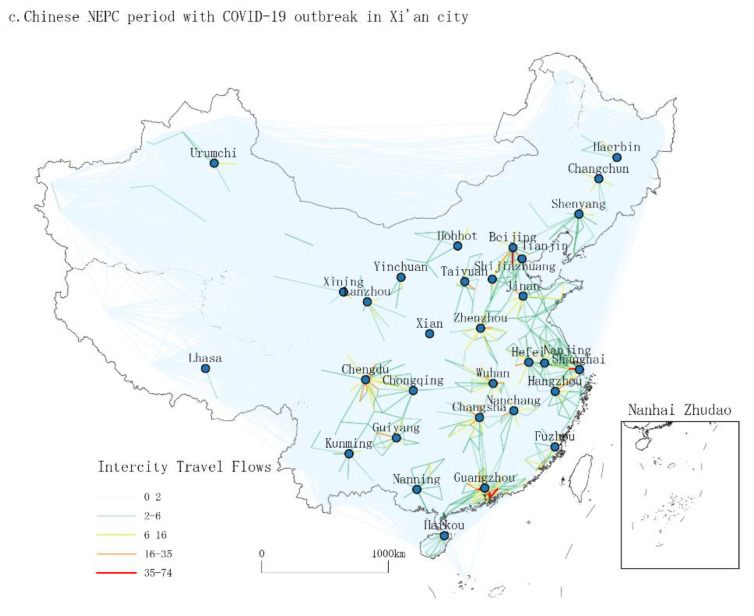
The figure shows the patterns of intercity travel flows in China during three periods before and after the COVID-19 epidemic. To clearly present the OD flow data, we averaged and scaled up the travel intensities. (**a**) Patterns of daily intercity travel before the outbreak of COVID-19 in December 2019; (**b**) patterns of daily intercity travel in NEPC period without COVID-19 outbreak in Xi’an in December 2020; (**c**) patterns of daily intercity travel in NEPC period with COVID-19 outbreak in Xi’an in December 2021.

From Figure 1, it is clear that the pattern of daily intercity interactions in China before the epidemic (Figure 1a) shows a “diamond” structure with Beijing, Shanghai, Guangzhou-Shenzhen, and Chengdu-Chongqing as the core (which can also include the periphery of Xi’an and Wuhan). The distribution of routes within and around the “diamond” is large and concentrated. This is consistent with the results of previous studies [8,43]. In contrast, the overall size, intensity, and distance of intercity travel in China during the NEPC period (Figure 1b) have decreased significantly, but the overall “diamond” structure has not changed. Comparing Figure 1b with Figure 1c, we can find that after the localized COVID-19 outbreak in Xi’an, travel in and around Xi’an almost stagnated, and the intensity of cross-flow between other cities also decreased. More notably, as a result of the Xi’an outbreak, intercity travel patterns in China no longer exhibit the traditional “diamond” structure, with intercity travel concentrated in small areas within the same province or neighboring cities. The figure above is an intuitive reflection of the negative inhibitory effect of the large-scale and localized epidemic events on intercity travel.

Then, we focus on the study period of this paper (9 December 2021 to 14 January 2022) to analyze the changes in intercity travel from the results of travel scale calculation. The results of $ITS_{change}$ numerically demonstrate the changes in human mobility in multiple locations across the country in the context of the Xi’an epidemic, which is the basis for quantitative analysis. Specifically, compared to the period of NEPC (P1), 56% of cities nationwide had a decrease of more than 5% in the travel index during the most severe period of the epidemic in Xi’an (P2), and 89% of cities had a decreasing trend in the intercity travel index. Among the top ten cities in terms of decline, Xi’an, the city hardest hit by the epidemic, experienced an 82% decline on the ITS index, which was largely due to the suppression of travel restriction policies under the epidemic. In addition, Shangluo, Xianyang, Weinan, and other cities with large decreases are close to Xi’an. A total of 9 of the top 10 $ITS_{Ck}$ rankings belong to the Shaanxi province jurisdiction. In addition, cities such as Pingliang, Qingyang, and Yuncheng also ranked high in terms of decline, although they belong to Gansu and Shanxi Province, adjacent to Shaanxi Province, and thus are also subject to the huge impact of the Xi’an epidemic. Exceptionally, the city of Fangchenggang, located in Guangxi Zhuang Autonomous Region and far away from Xi’an, ranks ninth in terms of intercity travel intensity decline since it also recorded new confirmed cases during the P2 phase.

In general, a large-scale epidemic would result in an overall decrease in travel intensity, but the macroscopic “diamond” structure remains the same. In contrast, localized epidemics may change overall travel patterns. As a result, we can confirm that small-scale localized outbreaks can lead to significant changes in intercity travel. An epidemic event in a single city will not only affect human activities within the city itself but will also have a radiating effect on neighboring and even more distant cities. It also shows that China’s attitude toward epidemic events is to react rapidly to outbreaks occurred and to prevent potential spread on time.

### 3.2. Temporal Variations of New COVID-19 Cases and Policy Index

The temporal variation of daily new cases is one way to represent the timeline of epidemic event development. At the provincial scale, the norm in China’s 31 provinces during the period of NEPC (P1) is that there are no daily new cases in most regions and there are only a small number of new cases in a few areas. However, once a localized epidemic in one place is not effectively controlled (e.g., the P2 phase of a major outbreak in Xi’an), it brings about a rebound in more areas (corresponding to the P3 period).

**Figure 2:**
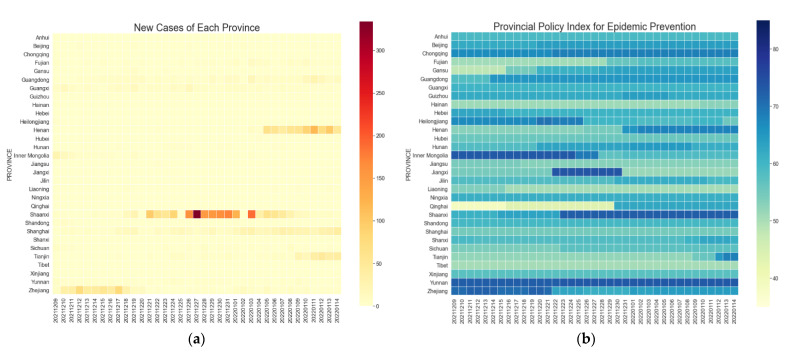
(**a**) Temporal variations of new cases by province; (**b**) temporal variations of policy index by province.

The visualization results of the provincial-scale epidemic case data and the epidemic prevention policy index are shown in Figure 2. It can be seen that the value of the policy index varies with local new cases in many regions, and there is a 45% correlation between them. During the P1 period of NEPC, most provinces had relatively lenient policies for epidemic prevention and control, with 65% of the provinces having PPI values below 60. During the P2 and P3 phases after the serious outbreak of the Xi’an epidemic, many provinces strengthened their epidemic policies, with more than half of the provinces having PPI values above 60, but differences existed in the time points of change. It is worth mentioning that quite a few regions have been maintaining strict epidemic strength even if they are far from Xi’an. For example, Beijing ranks among the top provinces in terms of policy strictness during the NEPC period, because Beijing, as the capital of China, needs to strictly prevent huge losses from the epidemic rebound. Another example is that PPI values in Yunnan province have been kept high during the study period, since Yunnan lies on the border of China and strict epidemic prevention measures need to be maintained to reduce the disease spread caused by the crowd movements and the importation of people from abroad.

In addition, at the city scale, the changes in *CPI* and *PPI* are consistent since PPI is a composite reflection of CPI, while CPI is a refined index considering the “number of epidemic risk areas”.

### 3.3. Influence Scope of the Localized Epidemic Event

The spatial lag analysis was conducted using the intercity travel scale reduction ($ITS_{Ck}$) of each city as the dependent variable, and the actual travel distance of Xi’an from other prefecture-level cities as the explanatory variable (denoted as xian_infl). The fitting results of the model are shown in Table 2. Compared with OLS, the model fit performance of SLM significantly improved. Both AIC and SC indices significantly decreased, and the log-likelihood results improved, which indicates that the spatial lag variables enhance the model fit results. For the regression coefficients, the coefficients of the spatial lag factor and the dependent variable were both significant at the 0.01 level. In other words, it is the severity of the Xi’an epidemic that brings about the different degrees of decline in ITS.

**Figure 3:**
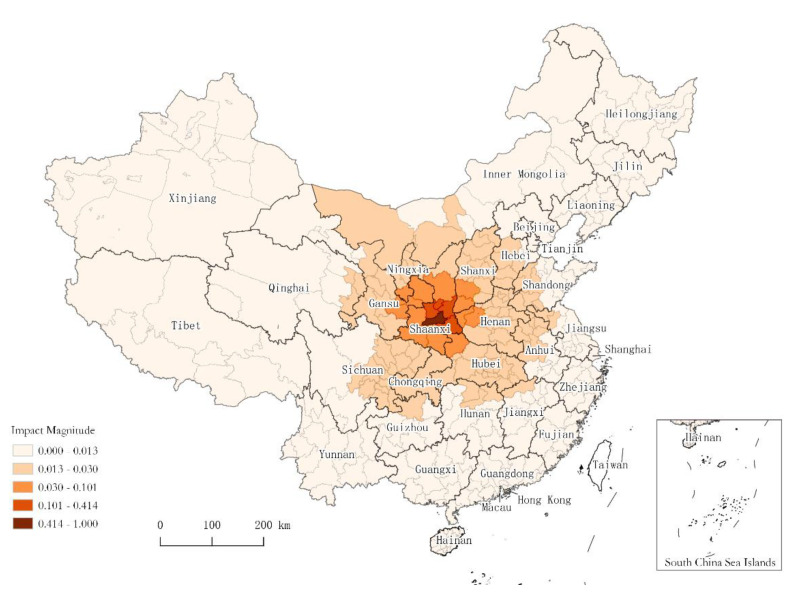
Influence scope of localized epidemic events in Xi’an.

Based on the above results, a visualization of how the Xi’an epidemic affects intercity travel in other cities is presented in Figure 3, using the administrative divisions of the cities as boundaries. In the figure, with Xi’an as the center of the circle, the impact of the Xi’an outbreak on intercity travel is found to radiate in a “ripple” pattern. The inhibitory effect on travel decreases with increasing distance, centered on the location of the outbreak. A closer analysis of the data shows that the localized epidemic in Xi’an has a broader and more severe impact on intercity travel in eastern cities than in western regions. This may be explained by the fact that eastern China tends to have regions with more plains, while the terrain west of Shaanxi Province gradually elevates. Moreover, the economy of eastern cities is relatively more developed than that of western cities, and intercity communication is more frequent in the east, making it easier for the epidemic to spread, and so the eastern region is hit more by the epidemic. In addition, it is found that some areas such as Aba Tibetan and Qiang Autonomous Prefecture in Sichuan Province, though not far from Xi’an in terms of actual geographic space, are less affected by the epidemic because they are located on the Qinghai-Tibetan plateau with high altitude and sparse population to hinder the spread of diseases.

The above analysis reveals a lesson: local events of a certain scale have an impact that cannot be underestimated, not only on the place where the event occurs but also on the wider area. As we can see, the analysis of urban events has important theoretical analysis and application value.

### 3.4. Relationship between Local COVID-19, Policy Index, and Intercity Travel

A quantitative analysis of the factors influencing intercity travel in the study period of P3 (5–14 January 2022) was conducted. During the P3 period, the local epidemic in Xi’an gradually recovered, while Dongguan, Kunming, Ningbo, and other cities experienced different levels of epidemics. It is suitable for a “many-to-many” evaluation of how the response policies of different epidemic events affect intercity population mobility. In this study, the traditional gravity model is modified and the regression results are shown in Table 3.

MODEL(0) is the primitive gravity model without the inclusion of epidemic case factors, epidemic policy factors, or heterogeneity variables. MODEL(1) incorporates three epidemic case indicators that visually describe the current status of the epidemic. MODEL(2) takes into account the epidemic prevention policy indices of the origin and destination. MODEL(3) builds on MODEL(2) and further considers two heterogeneity factors: “whether the two cities are adjacent to each other" and "whether the two cities belong to the same province”.

Firstly, comparing the goodness of fit of the four groups of models, the adjusted $R^{2}$ gradually increases with the addition of variables. Three groups of factors of $Covid_{status}$ in MODEL(1) exhibit different levels of significance. Among them, the number of new confirmed cases per day (New) and the number of existing confirmed cases (Existing) are significant at the 0.01 and 0.05 levels, respectively, for the suppression of trip intensity between two cities. Relatively, the effect of daily average new confirmed cases in the previous seven days (7d_avg_new) was not significant. In MODEL(2), there is a significant negative effect of the epidemic prevention policy index (*CPI*) on the origin and destination of intercity travel. That is, the stricter the city’s epidemic prevention policy, the less intercity crowd activity. The epidemic policy imposes restrictions on travel supply and demand, with fewer travel opportunities at the origin and less attractive destinations. In terms of the magnitude of the coefficients, the *CPI* at the destination appears to have a more significant effect than the *CPI* at the origin. From the perspective of travel utility, there are fewer travel options. In other words, people prefer to travel to places with relatively lenient prevention policies. In MODEL(3), the heterogeneity factors $SamePro_{ij}$ and $Adjacent_{ij}$ both bring a significant increase in $R^{2}$, indicating that the geographical location and spatial relationship between cities are indeed factors that cannot be ignored in intercity travel of the crowd. Cities close to each other have stronger travel intensity, and so do cities belonging to the same province. When the heterogeneity variable in MODEL(3) is $SamePro_{ij}$, the effect of $VAR*lnCPI_{i}$ and $VAR*lnCPI_{j}$ variables is significantly positive, indicating that the suppression effect of the CPI of two cities on intercity travel is stronger when they belong to the same province. Similarly, when the heterogeneity variable in MODEL(2) is $Adjacent_{ij}$, the coefficients of $VAR*lnCPI_{i}$ and $VAR*lnCPI_{j}$ variables are also significantly positive, indicating that the results of two cities being adjacent to each other are consistent with the results of two cities belonging to the same province, both of which deepen the control effect of urban epidemic prevention policies. In practical terms, that is because cities in the same province or adjacent to each other tend to have high economic activities and are more likely to adopt similar epidemic prevention policies. A change in the policy index correspondingly brings about a greater degree of change in intercity travel than in two cities that are farther apart.

The above analysis inspires us: when an event of a certain scale and influence occurs in a city, it is advisable to model not only the impact of the event itself on the city where the event occurred but also the consequences of the event from the perspective of the policies that respond to the event, which laterally reflect the nature of the event. At the same time, when analyzing the spillover effects of event factors that affect the region, one can focus on areas that are spatially or attribute-related to the place where the event occurred. This idea can also be extended to the impact analysis of other intercity linkages.

## 4. Conclusions and Discussions

Although much research has assessed the impact of the epidemic on crowd mobility from the perspective of epidemic cases, few studies have focused on the influence of variable localized epidemic patterns on mobility during the period of normalized epidemic prevention and control one or two years after the initial outbreak. The goal of this study is to compare and explain changes in intercity travel patterns before and during the NEPC period in China, and to explore the dynamic impact of small-scale localized epidemic events and related policies on intercity travel.

The conclusions of our work include: (1) Firstly, we compare the changes of intercity travel patterns in China before and after the outbreak of COVID-19 on the whole, and find that the large-scale epidemic in 2020 caused an overall decline in the intensity of Chinese intercity travel, but the “diamond” pattern of travel remained unchanged. Meanwhile, we estimate the heterogeneity of localized epidemic events on the reduction in population movement in different regions during the period of NEPC in China, using the epidemic in Xi’an, Shaanxi Province, China from 9 December 2021 to 14 January 2022 as an example. Localized epidemics are observed to have dramatic effects on intercity population movements and have a wide range of effects, changing the overall structure of intercity travel in China. (2) Secondly, we laterally portray localized city events from a policy perspective, which complements the visual description of city events. It is found that in the case of localized epidemic events in China, there is a synchronization between event occurrence and policy response. (3) The third, improved gravity model analysis shows that there is a significant negative correlation between both epidemic cases as well as the epidemic prevention policy index and intercity population mobility. This suggests that the policy index can effectively capture the negative impact of a catastrophic event on the economically relevant activities of a country or region. (4) Finally, we emphasize that spatial or attribute correlations between cities (e.g., two regions in the same province or two regions adjacent to each other) are also critical factors affecting the interaction strength in intercity travel analysis.

The analysis in this paper attempts to provide some insight into intercity travel research under the epidemic. Numerous related studies are devoted to digging into the conventional travel patterns over long periods of time or special occasions such as holidays [7,43,44], and there is a lack of fine-grained research on travel patterns on a daily basis. The advantages of massive location-based big data should be fully utilized to portray the intercity travel patterns of the crowd in a more detailed and timely manner with daily and weekly time scales. At the same time, more attention should be paid to the changes of intercity travel under the influence of emergencies, which can provide a reference for the allocation of traffic resources and policy control of unexpected events. Localized epidemics can be seen as events with significant impact in cities, which could have intricate effects on urban environments, populations, and management systems. Unlike planned activity events such as large sporting events and entertainment shows, the occurrence of catastrophic events such as epidemic events, extreme weather, and earthquakes is unpredictable. However, they all affect people’s real and online activities, from minor ones causing intracity traffic congestion to major ones causing intercity or nationwide economic exchange disruptions [45]. Therefore, the occurrence and possible effects of city events deserve attention.

In addition, in intercity interaction studies, numerous scholars have studied the influence of sociodemographic [46,47], urban industrial and economic [39], and urban infrastructure factors [33,48], but constraints of policies or measures are lacking. On the one hand, it is difficult to quantify policies because it is highly subjective. On the other hand, the benchmarks for policy formulation are heterogeneous due to the variation of different cities themselves. China’s epidemic prevention policies show us the non-negligibility of policy factors. However, it is a pending scientific issue to discuss how to portray the quantitative changes in travel due to specific measures. For example, it would be useful to analyze the extent to which specific policies and measures such as a seven-day quarantine of entry, a ban on large gatherings, and the closure of public places in a given area would each quantitatively change mobility in that place. In case the epidemic returns, such studies can provide valuable support for the scientific formulation of policies. Through rational policy design and arrangement, official governments can maximize the travel needs of people while strictly containing the spread of the epidemic. This is one of the directions that we will strive for in the future.

The major limitation of this paper is how to distinguish the reduction in subjective travel intentions from the reduction in travel due to policy restrictions. In this regard, based on the results of the SLM in Section 3.3, we selected 227 areas that were not affected by the epidemic cases in Xi’an for analysis. According to the results, epidemic cases and policy restrictions both bring about a decrease in the travel scale of people, but the policy restrictions are more convincing from the results of the goodness-of-fit parameter calculation. Although we cannot completely exclude the decrease in people’s subjective willingness to travel due to the increase in the number of epidemic cases, we consider its influence as a small percentage since we focus on the NEPC period in China, where the masses’ fear of disease has been significantly reduced. In addition, we have added epidemic cases as influential factors that directly characterize epidemic events in the gravity model, the results of which can reflect the subjective factor of travelers’ voluntary behaviors to some extent. In the future, it is necessary to combine rich social perception big data, questionnaire data, and interview data to better distinguish the effects of subjective and objective influencing factors on intercity travel.

## Figures and Tables

**Table 1:** Main variables and implications of COVID-19 case data and policy index.

Variables	Implications
Total	Cumulative number of confirmed cases
Cures	Cumulative number of cures
Deaths	Cumulative number of deaths
Existing	Number of existing confirmed cases
New	New confirmed cases
*7d_avg_new*	The average number of new cases per day in the previous seven days
Covid_status	Status of the daily epidemic in a city
PPI	Provincial policy control index for epidemic prevention
CPI	City-level policy control index for epidemic prevention

**Table 2:** Fitting results of OLS and SLM.

Parameters	SLM	OLS
R2	0.731	0.383
Log-likelihood	489.871	368.379
AIC	−973.742	−732.758
SC	−962.01	−724.936
Spatial lag factor	0.7846 *	
Constant	−0.0002	−0.0272 *
xian_infl	−0.1087 *	−0.2933 *

* Significant at 0.01.

**Table 3:** Regression results of the modified gravity model.

VARIABLES	MODEL(0)	MODEL(1)	MODEL(2)	MODEL(3)	
				***VAR: SamePro_ij_***	***VAR: Adjacent_ij_***
$lnP_{i}$	1.21 ***	1.27 ***	1.24 ***	1.28 ***	1.26 ***
$lnP_{j}$	1.28 ***	1.23 ***	1.29 ***	1.30 ***	1.25 ***
$lnD_{ij}$	−1.90 ***	−1.84 ***	−1.90 ***	−1.55 ***	−1.66 ***
$Existing_{i}$		−0.0051 **			
$Existing_{j}$		−0.0044 **			
$New_{i}$		−0.0132 ***			
$New_{j}$		−0.0101 ***			
$7d\_avg\_new_{i}$		−0.0104 *			
$7d\_avg\_new_{j}$		−0.0096			
$lnCPI_{i}$			−0.24 ***	−0.30 ***	−0.26 ***
$lnCPI_{j}$			−0.35 ***	−0.39 ***	−0.38 ***
$VAR\_lnCPI_{i}$				0.17 ***	0.13 ***
$VAR\_lnCPI_{j}$				0.21 ***	0.27 ***
$adjusted\;R^{2}$	0.663	0.672	0.680	0.743	0.725

*** significant at 0.01, ** significant at 0.05, * significant at 0.01. Notes: (1) subscript i denotes the departure city, subscript j denotes the destination city, and subscript ij indicates the relationship between the departure city and the destination city.

## Data Availability

Data may be made available upon reasonable request.

## Supplementary Materials

The following supporting information can be downloaded at: https://www.mdpi.com/article/10.3390/ijerph192114421/s1, Table S1: Basic statistics of Chinese cities.
