# Supplementary material for: Exploring the Impact of Localized COVID-19 Events on Intercity Mobility during the Normalized Prevention and Control Period in China
Source: Int J Environ Res Public Health. 2022 Nov 3;19(21):14421. doi: 10.3390/ijerph192114421 (PMC9656845; doi:10.3390/ijerph192114421)
Supplement: Supplementary file 1 [file ijerph-19-14421-s001.zip › ijerph-1959498-supplementary.pdf]

Table S1. Basic statistics of Chinese cities.

Variable Type	Variables	Variable Description	Average value	Standard deviation	Minimum value	Maximum value
Population	Population size	Year-end resident population (million)	447.2	332.1	20	3416
Economy	GDP	Gross regional product (billion yuan)	3213.6	4645.4	158	38156
	Practitioner	Total number of Employees (ten thousand)	57.9	90.6	3.4	791.3
	Public finance expenditures	Expenditure from the general budget of local finance (100 million yuan)	562.5	801.8	31.8	8179.3
	Number of industrial enterprises	Number of industrial enterprises above designated size (unit)	1249.1	1701.7	3	11042
	Average wages of staff and workers	The average salary of Staff and workers (Yuan)	78251.8	173110.1	44953	173205
	The growth rate of gross product	The growth rate of Gross Regional Product (%)	6.5	1.9	-3.6	11.8
	Tertiary industry	The proportion of tertiary industry in GDP (%)	49.2	8.2	28.3	83.5
Resources	Number of patent applications	Number of patent applications in cities	8177	18073.5	4	166609
	Resources of colleges and Universities	Number of regular institutions of higher learning	14052	31191	4	261502
	Medical resource	Number of doctors per 10,000	28	12.3	10	83
	Tourist attractions	Number of Excellent Grade (3A and above) Scenic Spots	30	23.9	0	201
Urban mobility	Annual passenger volume	Total water, land, and air passengers (100 million person-time)	381.7	1162.3	0.029	9632.5
	Annual cargo volume	Total land, water, and air freight (100 million tons)	6.5	29.4	0.002	416.6
Region level	administrative level	The administrative level of a city (municipality directly under the central Government -3, sub-provincial city -2, general city -1)	1.08	0.315	1	3
